# A incidência regional do tromboembolismo venoso no Brasil

**DOI:** 10.1590/1677-5449.000517

**Published:** 2017

**Authors:** Alan Vitor Ohki, Bonno van Bellen

**Affiliations:** 1 Hospital Beneficência Portuguesa – BP, Programa de Pós-Graduação Lato Sensu em Cirurgia Vascular Integrada, São Paulo, SP, Brasil.; 2 Hospital Beneficência Portuguesa – BP, Programa de Pós-Graduação Lato Sensu em Cirurgia Vascular Integrada, Serviço de Cirurgia Vascular Integrada, São Paulo, SP, Brasil.

**Keywords:** tromboembolismo venoso, trombose venosa, sazonalidade, clima

## Abstract

**Contexto:**

Vários estudos realizados em climas temperados sugerem que há uma variação na incidência de tromboembolismo venoso (TEV) de acordo com as estações climáticas. Entretanto, nenhum estudo foi feito comparando áreas de clima semiárido com áreas de clima temperado.

**Objetivos:**

Analisar se existe correlação entre a incidência do TEV em áreas de clima semiárido e de clima temperado no Brasil.

**Métodos:**

Foi feito um levantamento de dados retrospectivos de pacientes com diagnóstico de TEV no Sistema Único de Saúde de janeiro de 2011 a dezembro de 2014 provenientes dos seguintes estados com clima semiáridos: Alagoas, Ceará, Maranhão, Paraíba, Pernambuco, Piauí e Rio Grande do Norte, localizados na Região Nordeste do Brasil; e dos seguintes estados com clima temperado: Paraná, Santa Catarina e Rio Grande do Sul, localizados na Região Sul do Brasil. Os dados de variação climática foram obtidos do Instituto Nacional de Meteorologia e os dados populacionais do Instituto Brasileiro de Geografia e Estatística.

**Resultados:**

Houve correlação significativa na incidência de casos de TEV em regiões de temperaturas mais baixas (p < 0,001). A Região Sul apresentou temperaturas significativamente menores que as da Região Nordeste (p < 0,001) e apresentou número significativamente maior de casos de TEV do que a Região Nordeste (p < 0,001).

**Conclusão:**

Há mais casos de TEV em regiões de clima temperado, onde as temperaturas são mais baixas. No entanto, pouco ainda é conhecido na literatura sobre a flutuação sazonal e a incidência de TEV. Sendo assim, mais estudos são necessários nessa área.

## INTRODUÇÃO

O tromboembolismo venoso (TEV) é bastante prevalente no mundo, variando de 50 a 200 casos por 100.000 habitantes por ano^1^^-^ 4. A sazonalidade do TEV tem sido reportada em diversos estudos que foram realizados em áreas de clima temperado^1^^-^ 12. A maioria deles mostra haver maior prevalência em períodos de temperaturas mais baixas.

Entretanto, nenhum estudo foi feito comparando áreas de clima tropical semiárido com áreas de clima temperado. O presente estudo tem com objetivo analisar se existe correlação entre a incidência do TEV em áreas de clima semiárido e de clima temperado no Brasil.

## MÉTODOS

Foi feito um levantamento de dados retrospectivos de pacientes com diagnóstico de TEV no banco de dados do Sistema Único de Saúde (SUS)^13^. Foram selecionados pacientes atendidos de janeiro de 2011 a dezembro de 2014 e provenientes dos seguintes estados com clima semiárido: Alagoas, Ceará, Maranhão, Paraíba, Pernambuco, Piauí e Rio Grande do Norte, localizados na Região Nordeste do Brasil; e dos seguintes estados com clima temperado: Paraná, Santa Catarina e Rio Grande do Sul, localizados na Região Sul do Brasil.

Os dados climáticos das capitais dos estados supracitados foram obtidos no *site* oficial do Instituto Nacional de Meteorologia (Inmet)^14^ durante o mesmo período. Os dados populacionais foram obtidos no *site* oficial do Instituto Brasileiro de Geografia e Estatística (IBGE)^15^.

Os dados foram submetidos a análise estatística usando os testes de Spearman e de Mann-Whitney.

## RESULTADOS

Os valores descritivos das temperaturas dos estados avaliados estão na Tabela 1, na qual se pode observar que as medianas das temperaturas dos estados do sul foram mais baixas comparadas com as registradas nos estados do nordeste. Alguns meses não foram incluídos na tabela, pois não havia dados oficiais disponíveis (Tabela 1).

**Tabela 1:** Valores descritivos das temperaturas nos estados, expressas em graus Celsius.

**Estado**	**n**	**Média**	**Desvio padrão**	**Mediana**	**Mínimo**	**Máximo**
Região Nordeste						
Alagoas	48	25,21	1,13	25,32	23,31	27,26
Ceará	48	27,3	0,67	27,26	26,05	29,17
Maranhão	48	26,98	0,65	26,96	25,74	28,23
Paraíba	39	26,32	1,06	26,54	24,18	28,21
Pernambuco	48	25,6	1,12	25,79	23,47	27,58
Piauí	47	27,93	1,28	27,58	26,08	30,88
Rio Grande do Norte	48	26,44	1,02	26,68	24,64	28,04
Região Sul						
Paraná	46	18,02	2,73	18,26	12,83	22,78
Rio Grande do Sul	48	19,89	4,08	20,32	12,95	26,49
Santa Catarina	48	21,12	3,21	21,58	15,62	26,44

n = número de meses avaliados.

Os valores descritivos do número de casos de TEV para cada 100.000 habitantes nos estados avaliados estão na Tabela 2. Essa tabela demonstrou que as maiores medianas foram encontradas nos estados da Região Sul.

**Tabela 2:** Valores descritivos do número de casos de tromboembolismo venoso para cada 100.000 habitantes nos estados avaliados.

**Estado**	**n**	**Média**	**Desvio padrão**	**Mediana**	**Mínimo**	**Máximo**
Região Nordeste						
Alagoas	48	0,51	0,43	0,38	0,13	2,28
Ceará	48	0,95	0,35	0,93	0,44	1,68
Maranhão	48	0,26	0,08	0,27	0,08	0,46
Paraíba	48	0,45	0,14	0,44	0,13	0,74
Pernambuco	48	1,4	0,18	1,39	1,05	1,86
Piauí	48	0,4	0,13	0,38	0,13	0,74
Rio Grande do Norte	48	0,92	0,33	0,9	0,32	1,61
Região Sul						
Paraná	48	3,04	0,28	3,07	2,27	3,65
Rio Grande do Sul	48	2,95	0,29	2,96	2,4	3,62
Santa Catarina	48	2,57	0,22	2,58	2,13	3,26

n = número de meses avaliados.

Os valores descritivos das médias das temperaturas das regiões avaliadas estão na Tabela 3. Verificou-se que os estados do sul têm uma média de temperatura menor do que a dos estados do nordeste.

**Tabela 3:** Valores descritivos das médias das temperaturas das regiões, expressas em graus Celsius.

**Região**	**n**	**Média**	**Desvio padrão**	**Mediana**	**Mínimo**	**Máximo**
Nordeste	326	26,54	1,34	26,68	23,31	30,88
Sul	142	19,7	3,61	19,63	12,83	26,49

n = número de meses em que cada temperatura foi avaliada por estado.

**Figura 1:**
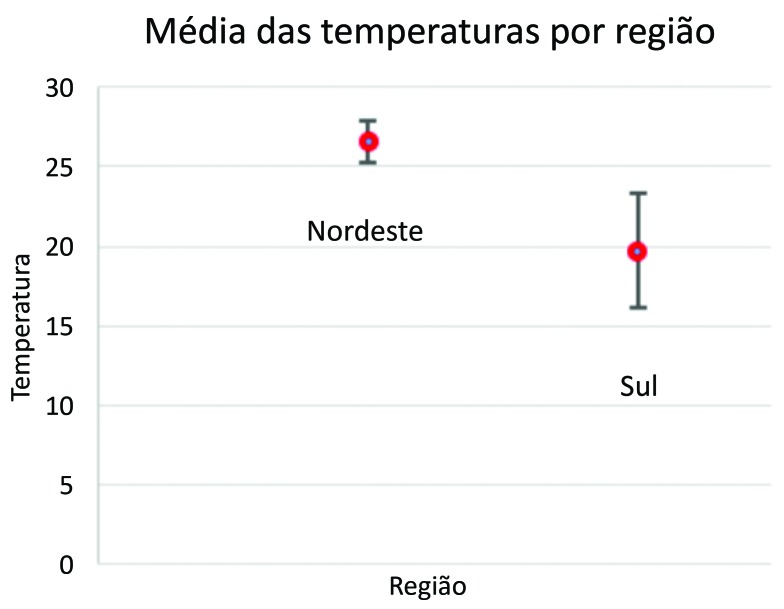
Variação da média de temperatura entre a Região Nordeste e a Região Sul em graus Celsius.

Através do teste não paramétrico de Mann-Whitney, observamos que há diferença significativa entre as regiões em relação à temperatura. A Região Sul apresentou temperaturas significativamente menores que as da Região Nordeste (p < 0,001) (Figura 1).

Os dados populacionais dos estados avaliados foram obtidos do IBGE. A população total da Região Sul era de 27.384.815 pessoas; e a da Região Nordeste era de 36.988.674 pessoas.

Os valores descritivos do número de casos de TEV para cada 100.000 habitantes nas regiões avaliadas estão na Tabela 4. A análise dessa tabela revelou que a mediana da Região Sul é maior do que a da Região Nordeste.

**Tabela 4:** Valores descritivos do número de casos de tromboembolismo venoso para cada 100.000 habitantes nas regiões avaliadas. n = número de meses de cada Estado avaliado.

**Região**	**n**	**Média**	**Desvio padrão**	**Mediana**	**Mínimo**	**Máximo**
Nordeste	336	0,70	0,46	0,53	0,08	2,28
Sul	144	2,86	0,33	2,82	2,13	3,65

n = número de meses de cada estado avaliado.

**Figura 2:**
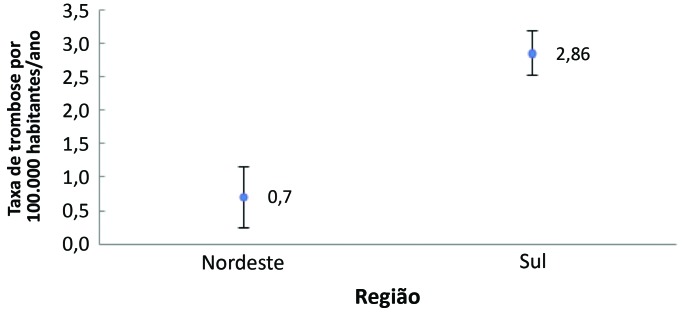
Casos de tromboembolismo venoso por 100.000 habitantes nas Regiões Nordeste e Sul.

Através do teste não paramétrico de Mann-Whitney, observamos que houve diferença significativa entre as regiões em relação ao número de casos de TEV. A Região Sul apresentou um número significativamente maior que a da região nordeste (p < 0,001) (Figura 2).

**Figura 3:**
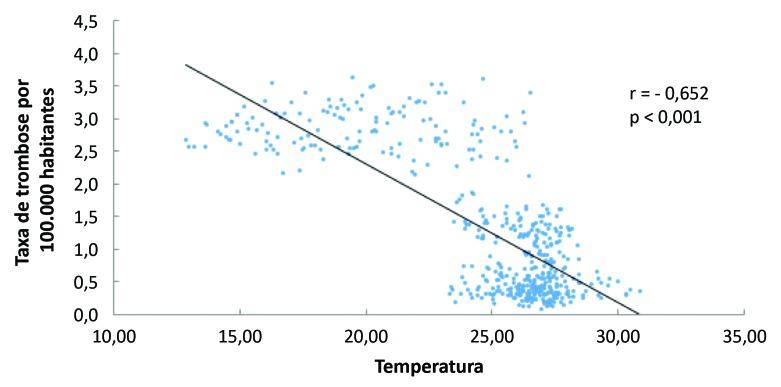
Correlação entre incidência de casos de tromboembolismo venoso para cada 100.000 habitantes e a temperatura em graus Celsius.

Através do coeficiente de correlação de Spearman, observamos que há correlação negativa e significativa entre temperatura e a taxa de TEV por 100.000 habitantes (r = -0,652; p < 0,001) (Figura 3).

## DISCUSSÃO

Na literatura, diversos trabalhos correlacionam casos de TEV e a variação climática. No trabalho de Stein et al.^5^, foi feito levantamento do banco de dados do *National Hospital Discharge Survey* durante o período de 1979 e 1999. Foram encontrados 7.682.000 pacientes com TEV, 2.457.000 com tromboembolismo pulmonar e 5.767.000 com TVP, e não foi evidenciada correlação entre variação sazonal e a incidência de TEV^5^.

Já o trabalho de Kleinfelder et al.^6^, que analisou 955 casos de TEV entre 1996 e 2003, constatou maior incidência nos meses quentes. Por outro lado, Brown et al.^7^, em análise retrospectiva de 37.336 casos durante 20 anos na Escócia, relataram maior incidência de TEV durante o mês mais frio (p < 0,0001). Resultados semelhantes foram obtidos por Gallerani et al.^8^ em estudo prospectivo de 1166 casos no Hospital Geral de Ferrara, Itália, que encontrou de maior incidência de TEV no inverno (p < 0,0001). Outros pesquisadores, como Ribeiro et al.^9^, Fink et al.^10^, Boulay et al.^11^ e Dentali et al.^12^, também constataram maior incidência de casos de TEV no inverno.

Existem alguns fatores e hipóteses que podem ser considerados. Durante os períodos mais quentes, as pessoas tendem a ficar mais sedentárias devido ao excesso de calor, promovendo maior imobilidade^16^. Já durante os meses mais frios, o desenvolvimento do TEV também pode ter relação com a diminuição da atividade física e com a vasoconstrição induzida pela baixa temperatura, que produz uma diminuição no fluxo sanguíneo dos membros inferiores^6^.

Ademais, as infecções do trato respiratório no inverno podem induzir a um estado de hipercoagulabilidade devido ao aumento dos níveis de fibrinogênio, o que também foi observado por Brown et al.^7^, Boulay et al.^12^ e Gallerani et al.^8^. Além disso, a diminuição do tempo de exposição à luz solar reduz a produção de melatonina e aumenta a coagulabilidade^6^.

Em condições frias, alguns fatores de coagulação estão aumentados *in vitro*, como a contagem de plaquetas e a agregação plaquetária, e também há diminuição do volume plasmático, o que aumenta a viscosidade do sangue e do fibrinogênio promovendo condições que aumentam os casos de trombose^11^.

Quanto ao consumo de líquidos, não há diferença no balanço hídrico em temperaturas mais quentes ou mais baixas. O consumo de líquidos é maior em temperaturas mais altas; porém, as perdas hídricas também são proporcionalmente maiores^17^.

No nosso estudo, os estados da Região Norte foram excluídos do trabalho devido à baixa amostragem. Na Região Nordeste, a amostragem é maior; no entanto, parece haver mais subnotificações de casos comparados aos dados da Região Sul, onde há mais recursos disponíveis na área da saúde. Os dados de incidência do TEV foram obtidos do SUS, que é o sistema oficial de saúde pública do Brasil.

Após análise estatística, houve correlação significativa na incidência de casos de TEV em temperaturas mais baixas (p < 0,001). Ou seja, quanto mais frio, maior é a incidência de TEV. A Região Sul apresenta temperaturas significativamente menores que as da Região Nordeste (p < 0,001) e apresenta número significativamente maior de casos de TEV do que a Região Nordeste (p < 0,001).

## CONCLUSÃO

Existe maior incidência de TEV nos estados do sul do Brasil, onde as temperaturas são mais baixas. No entanto, pouco ainda é conhecido na literatura sobre a correlação entre a flutuação sazonal e a incidência de TEV. Sendo assim, mais estudos ainda são necessários nessa área.
