# Supplementary material for: A incidência regional do tromboembolismo venoso no Brasil
Source: J Vasc Bras. 2017 Jul-Sep;16(3):227–31. [Article in Portuguese] doi: 10.1590/1677-5449.000517 (PMC5868939; doi:10.1590/1677-5449.000517)
Supplement: Translation

# Translation: Regional incidence of venous thromboembolism in Brazil

**Authors:** Alan Vitor Ohki, Bonno van Bellen

**Affiliations:** 1 Hospital Beneficência Portuguesa – BP, Postgraduate Sensu Lato in Vascular Surgery Integrated, São Paulo, SP, Brazil.; 2 Hospital Beneficência Portuguesa – BP, Postgraduate Sensu Lato in Vascular Surgery Integrated, Integrated Vascular Surgery Service, São Paulo, SP, Brazil.

**Keywords:** venous thromboembolism, venous thrombosis, seasonality, climate

## Abstract

**Background:**

Several studies conducted in areas with temperate climates have suggested that the incidence of venous thromboembolism (VTE) varies depending on seasonal climatic fluctuations. However, no studies have been conducted comparing areas with semi-arid climate with areas with temperate climates.

**Objectives:**

To analyze whether there is a correlation between the incidence of VTE in areas with semi-arid climates and areas with temperate climates in Brazil.

**Methods:**

Retrospective data were acquired for patients diagnosed with VTE from January 2011 to December 2014 from the Brazilian National Health Service for the following Brazilian states that have semi-arid climates: Alagoas, Ceará, Maranhão, Paraíba, Pernambuco, Piauí, and Rio Grande do Norte (all located in the Northeast Region of Brazil); and the following states with temperate climates: Paraná, Santa Catarina, and Rio Grande do Sul (all located in the South Region of Brazil). Data on climatic variation were obtained from the National Meteorological Institute and population data were obtained from the Brazilian Institute of Geography and Statistics.

**Results:**

There was a significant correlation in the incidence of VTE cases in areas with lower temperatures (p < 0.001). The South Region exhibited significantly lower temperatures than the Northeast Region (p < 0.001) and had a significantly higher number of cases of VTE than the Northeast Region (p < 0.001).

**Conclusions:**

There are more cases of VTE in areas with temperate climates, where temperatures are lower. However, there is still little information in the literature on seasonal fluctuations and incidence of VTE. More studies of this subject are needed.

## INTRODUCTION

The global prevalence of venous thromboembolism (VTE) is very high, ranging from 50 to 200 cases per 100,000 inhabitants per year.^1^^-^ 4 Seasonality of VTE incidence has been reported in various studies conducted in areas with temperate climates.^1^^-^ 12 The majority of them show that prevalence is higher during periods of lower temperatures.

However, no studies have been conducted that compare areas with semi-arid tropical climates with areas that have temperate climates. The objective of this study was to analyze whether there is a correlation between the incidence of VTE in areas with semi-arid climates and areas with temperate climates in Brazil.

## METHODS

Retrospective data were acquired for patients diagnosed with VTE from the Brazilian National Health Service (SUS - Sistema Único de Saúde).^13^ Patients were selected who had been diagnosed between January 2011 and December 2014 in the following Brazilian states that have semi-arid climates: Alagoas, Ceará, Maranhão, Paraíba, Pernambuco, Piauí, and Rio Grande do Norte (all located in the Northeast Region of Brazil); and the following states with temperate climates: Paraná, Santa Catarina, and Rio Grande do Sul (all located in the South Region of Brazil).

Climatic data for the capital cities of each of the states listed were obtained from the official National Meteorological Institute (Inmet - Instituto Nacional de Meteorologia),^14^ covering the same period. Population data were obtained from the official Brazilian Institute of Geography and Statistics’ (IBGE - Instituto Brasileiro de Geografia e Estatística).^15^

Data were subjected to statistical analysis using the Spearman and Mann-Whitney tests.

## RESULTS

Table 1 lists descriptive statistics for the temperatures of the states investigated, showing that the median temperatures in the Southern states were lower than the temperatures recorded in the states in the Northeast. Data for some months were not included in the table because official figures were not available (Table 1).

**Table 1:** Descriptive statistics for temperatures by state, in degrees Celsius.

**Region/State**	**n**	**Mean**	**Standard deviation**	**Median**	**Minimum**	**Maximum**
Northeast Region						
Alagoas	48	25.21	1.13	25.32	23.31	27.26
Ceará	48	27.3	0.67	27.26	26.05	29.17
Maranhão	48	26.98	0.65	26.96	25.74	28.23
Paraíba	39	26.32	1.06	26.54	24.18	28.21
Pernambuco	48	25.6	1.12	25.79	23.47	27.58
Piauí	47	27.93	1.28	27.58	26.08	30.88
Rio Grande do Norte	48	26.44	1.02	26.68	24.64	28.04
South Region						
Paraná	46	18.02	2.73	18.26	12.83	22.78
Rio Grande do Sul	48	19.89	4.08	20.32	12.95	26.49
Santa Catarina	48	21.12	3.21	21.58	15.62	26.44

n = number of months analyzed.

Table 2 lists descriptive statistics for the number of cases of VTE per 100,000 inhabitants in the states investigated. This table shows that the highest median values were for the states in the South Region.

**Table 2:** Descriptive statistics for number of cases of venous thromboembolism per 100,000 inhabitants of the states investigated.

**Region/State**	**n**	**Mean**	**Standard deviation**	**Median**	**Minimum**	**Maximum**
Northeast Region						
Alagoas	48	0.51	0.43	0.38	0.13	2.28
Ceará	48	0.95	0.35	0.93	0.44	1.68
Maranhão	48	0.26	0.08	0.27	0.08	0.46
Paraíba	48	0.45	0.14	0.44	0.13	0.74
Pernambuco	48	1.4	0.18	1.39	1.05	1.86
Piauí	48	0.4	0.13	0.38	0.13	0.74
Rio Grande do Norte	48	0.92	0.33	0.9	0.32	1.61
South Region						
Paraná	48	3.04	0.28	3.07	2.27	3.65
Rio Grande do Sul	48	2.95	0.29	2.96	2.4	3.62
Santa Catarina	48	2.57	0.22	2.58	2.13	3.26

n = number of months analyzed.

Table 3 lists descriptive statistics for average temperature in each Region (Northeast or South), showing that the states in the South had a lower mean temperature than the states in the Northeast.

**Table 3:** Descriptive statistics for mean temperatures of each Region, in degrees Celsius.

**Region**	**n**	**Mean**	**Standard deviation**	**Median**	**Minimum**	**Maximum**
Northeast	326	26.54	1.34	26.68	23.31	30.88
South	142	19.7	3.61	19.63	12.83	26.49

n = number of months for which temperature was analyzed in the states in each region.

The Mann-Whitney nonparametric test revealed a significant difference in temperature between the two regions. The South Region had significantly lower temperatures than the Northeast Region (p < 0.001) (Figure 1).

**Figure 1:** Variation in mean temperature in the Northeast Region and the South Region, in degrees Celsius.

Population data for the states investigated were obtained from IBGE. The total population of the South Region was 27,384,815 people and the total for the Northeast Region was 36,988,674 people.

Table 4 lists descriptive statistics for the number of cases of VTE per 100,000 inhabitants by Region. This table shows that the median is higher in the South Region than in the Northeast Region.

**Table 4:** Descriptive statistics for number of cases of venous thromboembolism per 100,000 inhabitants in each Region.

**Region**	**n**	**Mean**	**Standard deviation**	**Median**	**Minimum**	**Maximum**
Northeast	336	0.70	0.46	0.53	0.08	2.28
South	144	2.86	0.33	2.82	2.13	3.65

n = number of months for which cases were analyzed in the states in each region.

The Mann-Whitney nonparametric test revealed a significant difference between the Regions in terms of the number of cases of VTE. The South Region had a significantly higher number than the Northeast Region (p < 0.001) (Figure 2).

**Figure 2:** Cases of venous thromboembolism per 100,000 inhabitants in the Northeast and South Regions.

The Spearman’s correlation coefficient revealed a negative and significant correlation between temperature and rate of VTE per 100,000 inhabitants (r = -0.652; p < 0.001) (Figure 3).

**Figure 3:** Correlation between incidence of cases of venous thromboembolism per 100,000 inhabitants and temperature in degrees Celsius.

## DISCUSSION

Several studies in the literature correlate VTE cases with climatic variation. Stein et al.^5^ conducted a study analyzing data from the National Hospital Discharge Survey database covering the period from 1979 to 1999. They analyzed 7,682,000 cases of patients with VTE, 2,457,000 with pulmonary thromboembolism, and 5,767,000 with DVT, but did not find any correlation between seasonal variation and incidence of VTE.^5^

However, Kleinfelder et al.^6^ analyzed 955 cases of VTE from 1996 to 2003 and detected a higher incidence during hot months. In contrast, Brown et al.^7^ performed a retrospective analysis of 37,336 cases over 20 years in Scotland, reporting a higher incidence of VTE during the coldest month (p < 0.0001). Similar results were observed by Gallerani et al.^8^ in a prospective study of 1166 cases at the Hospital Geral de Ferrara, in Italy, where VTE incidence was higher in the winter (p < 0.0001). Other researchers, such as Ribeiro et al.,^9^ Fink et al.,^10^ Boulay et al.,^11^ and Dentali et al.,^12^ also reported higher incidence rates of VTE cases in the winter.

There are certain factors and hypotheses that can be considered with relation to these observations. During hotter periods, people tend to be more sedentary because of excess heat, increasing immobility.^16^ During colder months, development of VTE may also be related to reduced physical activity and to vasoconstriction induced by low temperatures, which reduces blood flow in the lower limbs.^6^

Additionally, respiratory tract infections during the winter may induce a hypercoagulable state due to increased fibrinogen levels, which was also observed by Brown et al.,^7^ Boulay et al.,^12^ and Gallerani et al.^8^ Furthermore, less time exposed to sunlight causes reduced melatonin production and increases coagulability.^6^

In cold conditions, some coagulation factors are elevated in vitro, such as platelet counts and platelet aggregation rates, while there is also a reduction in plasma volume, which increases the viscosity of the blood and of fibrinogen, providing conditions that increase the number of thrombosis cases.^11^

With regard to liquid consumption, there is no difference in fluid equilibrium between hotter and cooler temperatures. Liquid consumption is higher in hotter temperatures, but liquid losses are also proportionally higher.^17^

In our study, states in the North Region of Brazil were excluded from the analysis because of a lack of sample data. Sampling rates are higher in the Northeast Region, but it does appear that there is still a higher rate of under-notification of cases than in the data for the South Region, where there are more resources available for health care. Data on the incidence of VTE were obtained from the SUS, which is Brazil’s official public health system.

After statistical analysis, there was a significant correlation between incidence of VTE cases and lower temperatures (p < 0.001). Specifically, the colder the temperature, the higher the incidence of VTE. The South Region has significantly lower temperatures than the Northeast Region (p < 0.001) and had a significantly higher number of cases of VTE than the Northeast Region (p < 0.001).

## CONCLUSIONS

The incidence of VTE is higher in the states in the South of Brazil, where temperatures are lower. However, there is still little information in the literature on the correlation between seasonal fluctuation and incidence of VTE. More studies of this subject are therefore needed.
